# The Min System Disassembles FtsZ Foci and Inhibits Polar Peptidoglycan Remodeling in Bacillus subtilis

**DOI:** 10.1128/mBio.03197-19

**Published:** 2020-03-17

**Authors:** Yuanchen Yu, Jinsheng Zhou, Felix Dempwolff, Joshua D. Baker, Daniel B. Kearns, Stephen C. Jacobson

**Affiliations:** a Department of Chemistry, Indiana University Bloomington, Indiana, USA; b Department of Biology, Indiana University Bloomington, Indiana, USA; University of Michigan—Ann Arbor

**Keywords:** FtsZ, MinD, microfluidics, growth, cell division, peptidoglycan

## Abstract

A microfluidic system coupled with fluorescence microscopy is a powerful approach for quantitative analysis of bacterial growth. Here, we measure parameters of growth and dynamic localization of the cell division initiation protein FtsZ in Bacillus subtilis. Consistent with previous reports, we found that after division, FtsZ rings remain at the cell poles, and polar FtsZ ring disassembly coincides with rapid Z-ring accumulation at the midcell. In cells mutated for *minD*, however, the polar FtsZ rings persist indefinitely, suggesting that the primary function of the Min system is in Z-ring disassembly. The inability to recycle FtsZ monomers in the *minD* mutant results in the simultaneous maintenance of multiple Z-rings that are restricted by competition for newly synthesized FtsZ. Although the parameters of FtsZ dynamics change in the *minD* mutant, the overall cell division time remains the same, albeit with elongated cells necessary to accumulate a critical threshold amount of FtsZ for promoting medial division. Finally, the *minD* mutant characteristically produces minicells composed of polar peptidoglycan shown to be inert for remodeling in the wild type. Polar peptidoglycan, however, loses its inert character in the *minD* mutant, suggesting that the Min system not only is important for recycling FtsZ but also may have a secondary role in the spatiotemporal regulation of peptidoglycan remodeling.

## Abstract (precis)

Many bacteria grow and divide by binary fission in which a mother cell divides into two identical daughter cells. To produce two equally sized daughters, the division machinery, guided by FtsZ, must dynamically localize to the midcell each cell cycle. Here, we quantitatively analyzed FtsZ dynamics during growth and found that the Min system of Bacillus subtilis is essential to disassemble FtsZ rings after division. Moreover, a failure to efficiently recycle FtsZ results in an increase in cell size. Finally, we show that the Min system has an additional role in inhibiting cell wall turnover and contributes to the “inert” property of cell walls at the poles.

## INTRODUCTION

Bacillus subtilis is a Gram-positive rod-shaped bacterium that grows and divides by a process called binary fission, in which cells increase in mass and divide into two daughters of roughly equal size. During growth, the cell elongates by inserting new peptidoglycan into the lateral cell wall (1). As biomass increases, replication of the chromosome is initiated, and the chromosomes segregate such that the bulk of the nucleoids become evenly spaced within the cytoplasm (2). Cell division is initiated near the geometric midpoint of the cell, where peptidoglycan synthesis is reoriented inward toward the cytoplasm to build a septum and complete cytokinesis (3). Medial positioning of cell division ensures that the septum forms between the two nucleoids, guaranteeing that each daughter receives one copy of the chromosome. Although the mechanisms governing growth and cell division-site selection are complex, one of the first factors involved in cell division is the protein FtsZ.

FtsZ is a homolog of eukaryotic tubulin and exists in one of two different states in the cytoplasm, either as soluble monomers or in long filamentous polymers called protofilaments (4, 5). The two states rapidly interchange as protofilaments dynamically travel by a process called “treadmilling” in which FtsZ monomers are added to one end and lost from the other (4, 6–11). Treadmilling protofilaments form on the cytoplasmic facing of the membrane and coalesce into a bright focus called the Z-ring at the nascent site of cell division (12–14). Once mature, the Z-ring recruits a transmembrane complex of proteins known as the divisome that synthesizes peptidoglycan on the exterior of the cell (15, 16). The Z-ring constricts, either on its own or aided by divisome-directed peptidoglycan synthesis, until the septum is complete, resulting in cytokinesis (17, 18). Thus, FtsZ is both dynamic and (seemingly) static when concentrated at the site of cell division, and one of the first factors to be recognized as involved in controlling FtsZ dynamics and localization is the Min system.

The Min system was first discovered in Escherichia coli in the form of a mutant that produced minicells at high frequency (19). Minicells are small, metabolically active, spherical bodies that lack DNA and arise when cell division occurs not at the midcell but, rather, near one cell pole. Polar division was attributed to a mislocalization of FtsZ rings and the recruitment of the same machinery that would ordinarily promote medial septation (20). The mutation responsible for minicell formation was mapped to a genetic locus encoding the membrane-associated ATPase MinD and the FtsZ inhibitor MinC (21, 22). MinD associates with the membrane through an amphipathic helix, and MinD recruits and activates MinC by direct interaction (23–30). MinC binds to the C terminus of FtsZ and destabilizes the FtsZ ring (31–35). In E. coli, the activity of the MinCD complex is dynamically restricted to the polar region by oscillation of the MinE antagonist (36–40). In B. subtilis, however, the activity of the MinCD complex is statically restricted to membranes with high curvature by MinJ/DivIVA, such that the entire 4-protein complex assembles at the invaginating nascent division plane and remains at the nascent cell poles after division (41–46).

Here, we use fluorescence microscopy and microfluidics to quantitatively measure parameters of B. subtilis FtsZ dynamics and cell division under the condition of chemostatic growth for extended periods of time (47–52). The automated poly(dimethylsiloxane) microfluidic system comprises a pneumatically actuated array of 600 channels having widths of 1.0 to 1.6 μm and heights of 1.0 μm to actively trap bacterial cells (52). Integrated pumps and valves perform on-chip fluid and cell manipulations that provide dynamic control of cell loading and nutrient flow, and the channel array confines bacterial growth to a single dimension, facilitating high-resolution, time-lapse imaging and tracking of individual cells over multiple generations. In wild-type cells, we find that Z-rings persist for a period longer than the generation time because Z-rings transiently remain at the cell poles following septum completion. We further show that the primary function of the Min system is Z-ring disassembly such that in the absence of Min, Z-rings persist longer than the duration of the experiment. Indefinite Z-ring persistence results in cells with multiple Z-rings per compartment. Moreover, we show that *min* mutant cells are elongated because of a failure to recycle monomers and that competition between multiple Z-rings necessitates a larger FtsZ pool. Finally, we provide evidence that the B. subtilis Min system also inhibits cell wall turnover, particularly at the poles of the cell.

## RESULTS

### The Min system is required for FtsZ ring disassembly.

**FIG 1:**
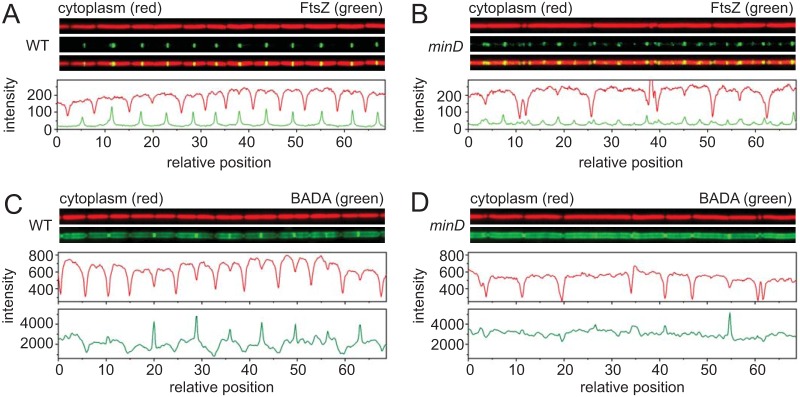
Microfluidic analysis of growth and division in the wild type and in *min* mutants of Bacillus subtilis. Snapshot fluorescence microscopy of a microfluidic channel was performed for the wild type (WT) (A and C) and *minD* mutants (B and D) growing at steady state. (A and B) Fluorescence microscopy of the wild type (DK5133) (A) and a *minD* mutant (DK5155) (B) in a microfluidic channel expressing cytoplasmic mCherry protein (falsely colored red; top) or mNeongreen-FtsZ (falsely colored green; middle) and an overlay of the two (bottom). Graphs present results from a quantitative analysis of mCherry fluorescence intensity (red line) and mNeongreen fluorescence intensity (green line) and match the fluorescence microscopy images immediately above. All images are reproduced at the same magnification. (C and D) Fluorescence microscopy of a microfluidic channel for the wild type (DK4394) (C) and a *minD* mutant (DK4407) (D) growing at steady state. Images represent cytoplasmic mCherry protein (falsely colored red; top) and peptidoglycan stained with BADA (falsely colored green; bottom). Graphs present results from a quantitative analysis of mCherry fluorescence intensity (red) and BADA fluorescence intensity (green) and match the fluorescence microscopy images immediately above. All images are reproduced at the same magnification.

**FIG 2:**
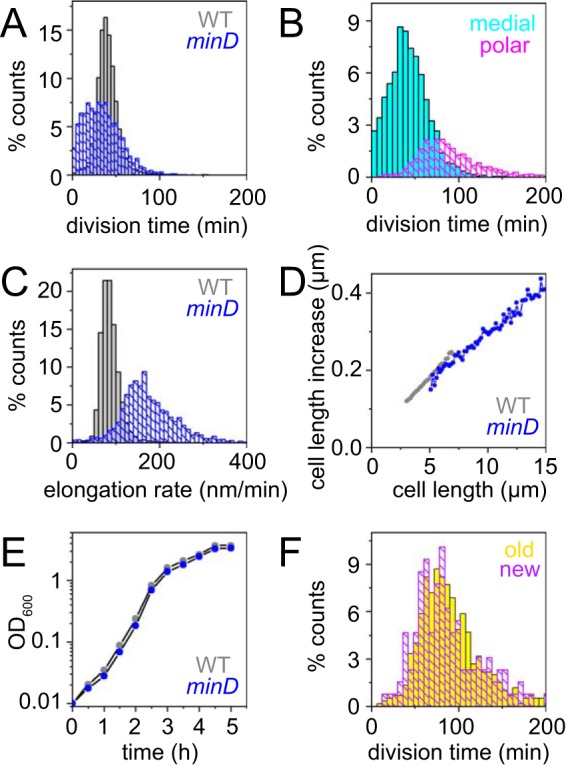
Cells mutated for the Min system divide faster than wild-type cells. (A) A histogram of the division time of individual cells of the wild-type strain (gray) and a *minD* mutant (blue) measured by microscopic analysis. Division events were defined by a local 20% decrease in mCherry (cytoplasmic) fluorescence intensity below a threshold value. More than 3,000 division events were counted per data set. Minicells were excluded from the division time analysis as once they are formed, minicells never divide. (B) A histogram of division time of individual cells of the *minD* mutant from the data set represented in panel A to separately determine the time elapsed between the medial and polar divisions. The time between medial division events (cyan) was determined as the time between division events that gave rise to two mother cells. The time between polar divisions (magenta) was determined to be the time between the formation of a cell pole and the formation of a division plane at that pole to give rise to a minicell. (C) Cell elongation rates were measured as the rate at which the cell poles moved apart from one another in the wild-type strain (gray) and a *minD* mutant (blue). The growth rates of over 2,500 cells were measured for each strain. Minicells were excluded from the elongation rate analysis as once they are formed, minicells do not elongate. (D) Data from panel C were replotted as the instantaneous increase in cell length per total length of the cell observed. (E) Growth curve of the wild-type strain (gray) and a *minD* mutant (blue) growing in highly agitated LB broth at 37°C. Optical density at 600 nm (OD_600_) was measured with a spectrophotometer (600-nm wavelength). (F) Frequency histogram of the division time that gives rise to minicells at either the old cell pole (yellow) or the new cell pole (violet). Old cell poles were defined as the poles that had last experienced a polar division event. New cell poles were defined as the poles that had not previously experienced a polar division event. The wild-type strain (DK5133) and a *minD* mutant (DK5155) were used to generate all of the data in this figure.

Quantitative microscopic analysis of cell growth and division on agarose pads is restricted by the limited number of generations that can be observed under batch conditions. To circumvent this problem, a microfluidic-based approach was undertaken to monitor steady-state chemostatic growth of B. subtilis over many generations (see Fig. S1 in the supplemental material). B. subtilis divides by septation (or plate formation) in which a division septum is formed first, and remodeling of the septal peptidoglycan occurs as a separate step that leads to indentation and cell separation (53–55). Thus, cell division events were conservatively defined as representing a spatial decrease in constitutively expressed cytoplasmic mCherry fluorescent signal that would indicate cellular indentation (Fig. 1A). Images were captured every 2 min, and the fluorescence intensity was measured along the length of the microfluidic channel. After inoculation into the microfluidic device, a period of roughly 3 h elapsed during which cells appeared to adjust to the growth conditions, and steady-state growth was maintained and monitored over the next 21 h (see Movie S1 in the supplemental material). Microscopic analysis indicated that the wild-type cell division time, defined as the time between two septum formation events, was 39 ± 12 min (Fig. 2A).

10.1128/mBio.03197-19.1 FIG S1 Diagram of the microfluidic device. Black lines indicate microfluidic channels. Open circles indicate media/reagent input/output reservoirs. Closed circles indicate the location of peristaltic valves. Three valves in parallel indicate a peristaltic pump. The location of the nanochannel array imaged by fluorescence microscopy is indicated by a red box. The same device was used previously as described in reference 52. Download FIG S1, PDF file, 0.4 MB. Copyright © 2020 Yu et al. 2020 Yu et al. This content is distributed under the terms of the Creative Commons Attribution 4.0 International license.

10.1128/mBio.03197-19.5 MOVIE S1 Wild-type growth in microfluidic channels. Constitutive cytoplasmic mCherry is falsely colored red. Images represent strain DK5133. Movies are 200 frames taken over 400 min at a rate of 1 frame/2 min. Download Movie S1, AVI file, 4.4 MB. Copyright © 2020 Yu et al. 2020 Yu et al. This content is distributed under the terms of the Creative Commons Attribution 4.0 International license.

**FIG 3:**
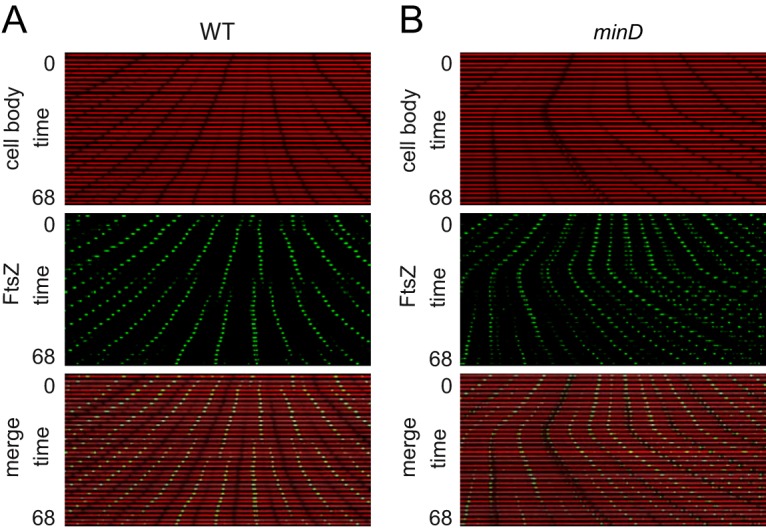
FtsZ foci remain at the poles indefinitely in the absence of Min. Data represent results of kymograph analysis of the wild-type strain (A) and a *minD* mutant (B) of cytoplasmic mCherry signal (falsely colored red; top) and mNeongreen-FtsZ intensity (falsely colored green; middle) and an overlay of the red and green channels (bottom). In each panel, a single microfluidic channel was followed in a series of stacked snapshots taken at 2-min intervals to assemble the kymograph. Polar Z-rings were persistent in the *minD* mutant (see Movie S4) but fluctuated in intensity. We note that decreases in *minD* polar Z-ring intensity often corresponded to polar cell division events that gave rise to minicells. All images are reproduced at the same magnification. The wild-type strain (DK5133) and a *minD* mutant (DK5155) were used to generate all of the data in this figure.

**FIG 4:**
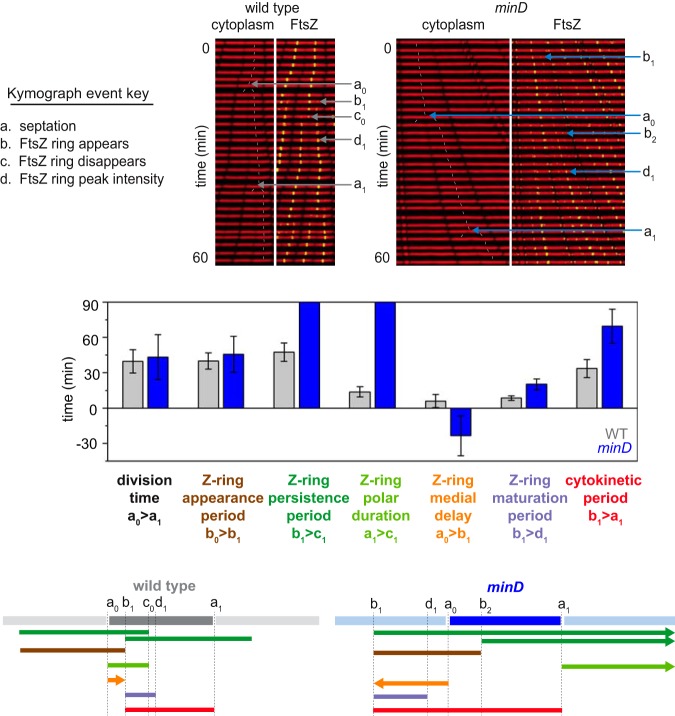
The cytokinetic period is longer than the cell division time of a *minD* mutant. (Top) Sample kymograph analysis of a microfluidic channel in which either the wild-type strain or a *minD* mutant was grown. Cytoplasmic mCherry signal is falsely colored red (left) and overlaid with mNeongreen-FtsZ (falsely colored green) (right). Events necessary for defining division parameters are indicated and labeled as follows: a, septation; b, appearance of a nascent Z-ring; c, disappearance of a Z-ring; d, FtsZ peak intensity achieved. Each event designation was given a number as follows: 0, preceding generation; 1, current generation; 2, subsequent generation. Thin white lines are included to indicate cell tracking and lineage analysis. (Middle) Graphs of 100 manually tracked wild-type (gray) and *minD* mutant (blue) mother cell division cycles presented as bars of average values and whiskers of standard deviations for the following parameters: division time (the time between cell septation events [i.e., between consecutive “a” events]); Z-ring appearance period (the time between the appearance of one Z-ring and another [i.e., between consecutive “b” events]), Z-ring persistence period (the time between the appearance of a Z-ring and the disappearance of that Z-ring [i.e., between consecutive “b” and “c” events]), Z-ring polar duration (the time between a septation event and the disappearance of the Z-ring resulting from that septation events [i.e., between consecutive “a” and “c” events]), Z-ring medial delay (the time between a septation event and the appearance of a Z-ring that will eventually give rise to the next medial division event [i.e., between an “a” event and a “b” event that will give rise to the next round of septation]), Z-ring maturation period (the time between Z-ring formation and when that Z-ring achieves peak local intensity [i.e., between consecutive “a” and “d” events]), and cytokinetic period (time between Z-ring formation and septation directed by that Z-ring [i.e., between a “b” event and the “a” event that is caused by that particular Z-ring]). The raw data representing the histogram for each bar are presented in Fig. S2. (Bottom) Time line representations of the various events indicated in the bar graph depicted in cartoon form, color coded to match the indicated parameter of like color above, and annotated with relevant events marked by the defining letters.

**FIG 5:**
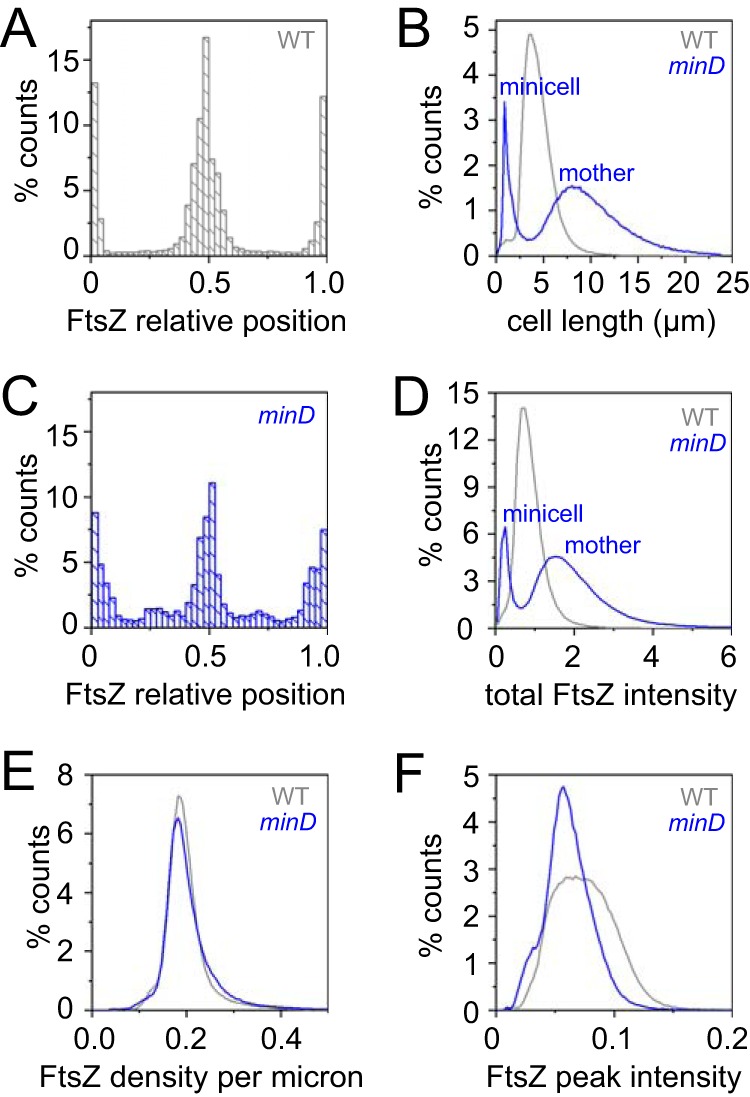
FtsZ density is constant, but FtsZ accumulations in Z-rings differ in the wild type and the *minD* mutant. (A) A frequency histogram of the location of snapshot peak FtsZ intensity plotted relative to total cell length for wild type. The poles of the cell have relative position values of 0 and 1.0, whereas the midcell has a value of 0.5. (B) A frequency histogram of cell length distribution of the wild-type strain (gray) and a *minD* mutant (blue). The *minD* mutant has two peaks, namely, a shorter peak corresponding to “minicells” and a longer peak corresponding to “mother” cells that have chromosomes and are capable of division. (C) A frequency histogram of the location of snapshot peak FtsZ intensity plotted relative to total cell length for *minD*. (D) A frequency histogram of total FtsZ fluorescence intensity per cell. For each frame, multiple line scans through the longitudinal axis of the cell was performed, and total FtsZ fluorescence intensity was measured by integrating the area under the line scans. (E) A frequency histogram of FtsZ density was produced by dividing the total fluorescence intensity by cell length for each individual. (F) A frequency histogram of peak FtsZ fluorescence intensity per cell. For each frame, multiple line scans through the longitudinal axis of the cell determined the location of peak fluorescence intensity, and peak fluorescence magnitude was recorded. Data represent wild-type strain distribution (gray) and *minD* strain distribution (blue). Measurements were taken for 9,000 cells of the growing wild-type strain (DK5133), amounting to over 30,000 measurements, and for 6,000 cells of the growing *minD* strain (DK5155), amounting to over 20,000 measurements, to generate the data in this figure.

Cell division is mediated by dynamic localization of the division initiation protein FtsZ (8, 9, 12, 14). FtsZ dynamics were monitored by fluorescence microscopy in a strain encoding an N-terminal fluorescent fusion of mNeongreen introduced at the native site in the chromosome (9). Images were captured every 2 min, and the fluorescence intensity magnitude of FtsZ was measured as a snapshot in the context of a fluorescent mCherry cytoplasmic signal (Fig. 1A). During steady-state growth, FtsZ appeared as a faint uniform cytoplasmic haze with bands of enhanced fluorescence intensity, and a kymograph was generated to track FtsZ dynamics in temporal relation to the cell body (Fig. 3A; see also Fig. 1A) (Movie S2). The FtsZ-ring appearance period, defined as the time between the appearance of one Z-ring and the appearance of the next, was found to be similar to that of the cell division time (Fig. 4; see also Fig. S2A). The Z-ring persistence period, defined as the time between the appearance and disappearance of a single FtsZ focus, was longer than the average period of cell division (Fig. 4; see also Fig. S2B), likely because FtsZ has been observed to be present transiently at the cell pole after cytokinesis (13, 46, 56). Consistent with previous observations, many cells exhibited a characteristic peak of FtsZ fluorescence intensity near the midcell to mediate division, but some cells instead exhibited peak fluorescence at the cell pole after division was complete (Fig. 5A). We conclude that FtsZ remains polarly localized after cytokinesis.

10.1128/mBio.03197-19.2 FIG S2 Data histograms of FtsZ parameters used to generate Fig. 4. A total of 100 cells were manually tracked and measured through a cell division cycle to generate the data for each panel. Data are presented as gray bars for the wild type (DK5133) and as blue bars for the *minD* mutant (DK5155). (A) Frequency histogram of the Z-ring appearance period, defined as the time between the appearances of one Z-ring and the next Z-ring. (B) Frequency histogram of the Z-ring persistence period defined by the time between the appearance of a Z-ring and the disappearance of that Z-ring. Note that no data are provided for the *minD* mutant as the Z-rings of a *minD* mutant did not disappear and the persistence period was effectively infinite. (C) Frequency histogram of the Z-ring polar duration defined as the time between a septation event and the disappearance of the Z-ring resulting from that septation event. Note that no data are provided for the *minD* mutant as the Z-rings of a *minD* mutant did not disappear and the polar duration period was effectively infinite. (D) Frequency histogram of the Z-ring medial delay defined as the time between a septation event and the appearance of the Z-ring that would eventually give rise to the next medial division event. Note that the value representing the Z-ring medial delay of the *minD* mutant was negative on average because the medial Z-ring that would eventually promote cell division had been formed in the preceding generation. (E) Frequency histogram of the Z-ring maturation period defined as the time between the Z-ring appearance and the time point at which that Z-ring achieved peak local intensity. (F) Frequency histogram of the cytokinetic period defined as the time between the Z-ring appearance and the septation directed by that Z-ring. Download FIG S2, PDF file, 0.8 MB. Copyright © 2020 Yu et al. 2020 Yu et al. This content is distributed under the terms of the Creative Commons Attribution 4.0 International license.

10.1128/mBio.03197-19.6 MOVIE S2 Wild-type growth in microfluidic channels with fluorescent FtsZ. Constitutive cytoplasmic mCherry is falsely colored red, and a mNeongreen-FtsZ is falsely colored green. Strain DK5133. Movies are 200 frames taken over 400 min at a rate of 1 frame/2 min. Download Movie S2, AVI file, 5.6 MB. Copyright © 2020 Yu et al. 2020 Yu et al. This content is distributed under the terms of the Creative Commons Attribution 4.0 International license.

To further explore Z-ring dynamics in the wild type, 100 cells were chosen at random, and a variety of parameters were manually measured. The Z-ring polar duration, defined as the time between septation and the disappearance of the Z-ring (Fig. 4; see also Fig. S2C), was longer than and overlapped the Z-ring medial delay (Fig. 4; see also Fig. S2D), defined as the time between septation and the formation of a new Z-ring. Thus, wild-type cells transiently experienced multiple FtsZ rings per compartment. Moreover, dissolution of the polar Z-ring coincided with the Z-ring maturation period (Fig. 4; see also Fig. S2E), defined as the time from first appearance of the medial Z-ring until maximum Z-ring fluorescence intensity, as FtsZ subunits were redistributed from the pole to the midcell. Finally, the cytokinetic period, defined as the time between Z-ring appearance and cell division, was approximately 33 ± 8 min (Fig. 4; see also Fig. S2F), which, when added to the Z-ring medial delay of 6 ± 6 min, ultimately produced a value similar to the FtsZ appearance period of 40 ± 7 min and the cell division time of 39 ± 12 min (Fig. 4). We conclude that the dynamic parameters of FtsZ are consistent with a regular cell division period despite the transient localization of the Z-ring at the poles.

One mechanism that governs FtsZ localization is the Min system (20, 21, 57). To explore the consequences of disruption of the Min system quantitatively, a mutation was introduced in the gene encoding MinD, the membrane-localized activator of the FtsZ inhibitor protein MinC, and the *minD* mutant was monitored during growth in microfluidic channels (Fig. 1B). Consistent with a *min* phenotype, the *minD* mutant produced two different cell types: cells that were longer than wild-type cells and very short minicells (Fig. 5B; see also Movie S3) (19, 21, 22, 58). Cells mutated for MinD produced multiple FtsZ foci (59) (Fig. 1B; see also Movie S4), and kymograph analysis indicated that while the FtsZ-ring fluorescence intensity fluctuated, the persistence period was indefinite and the fluorescence did not disappear during the time course of observation (Fig. 3B). Moreover, a greater proportion of *minD* mutant cells exhibited peak FtsZ fluorescence intensity at the poles (Fig. 5C). We conclude that when a cell divides, the FtsZ focus is split into the two daughter cells. In the wild type, the polar FtsZ ring is transient due to antagonism by Min, but in the absence of Min, the ring persists. Our data support models in which the primary function of the Min system during division is to promote FtsZ ring disassembly (23, 46, 60).

10.1128/mBio.03197-19.7 MOVIE S3 *minD* mutant growth in microfluidic channels. Constitutive cytoplasmic mCherry is falsely colored red. Images represent strain DK5155. Movies are 200 frames taken over 400 min at a rate of 1 frame/2 min. Download Movie S3, AVI file, 4.4 MB. Copyright © 2020 Yu et al. 2020 Yu et al. This content is distributed under the terms of the Creative Commons Attribution 4.0 International license.

10.1128/mBio.03197-19.8 MOVIE S4 *minD* mutant growth in microfluidic channels with fluorescent FtsZ. Constitutive cytoplasmic mCherry is falsely colored red, and a mNeongreen-FtsZ is falsely colored green. Images represent strain DK5155. Movies are 200 frames taken over 400 min at a rate of 1 frame/2 min. Download Movie S4, AVI file, 5.7 MB. Copyright © 2020 Yu et al. 2020 Yu et al. This content is distributed under the terms of the Creative Commons Attribution 4.0 International license.

### The Min system maintains cell size by recycling FtsZ.

**FIG 6:**
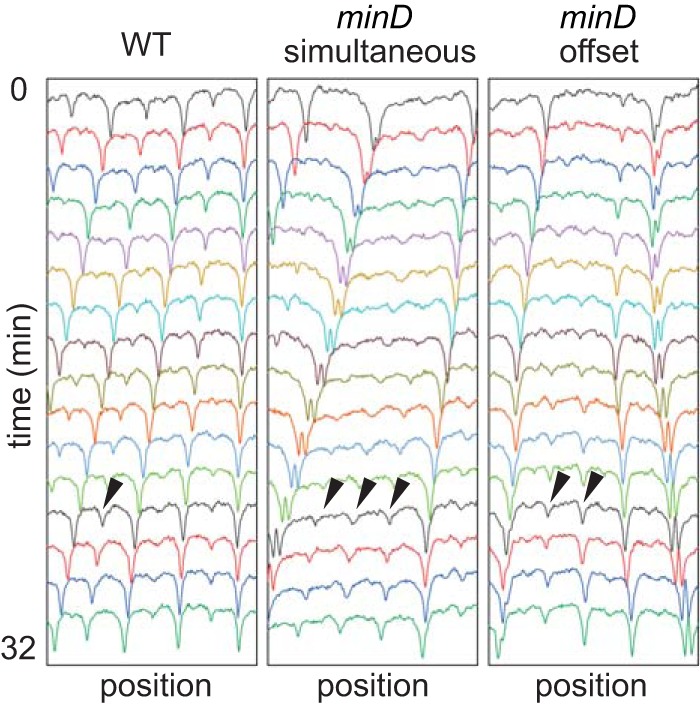
Some cells of a *minD* mutant experience multiple simultaneous divisions. Kymograph analysis of the cytoplasmic mCherry fluorescence intensity of single channels of the wild-type strain (left) or *minD* mutant (middle and right) was performed. Wild-type cells experience regular division once per cell cycle (arrowhead, left panel), but multiple simultaneous (arrowheads, middle panel) or slightly offset (arrowheads, right panel) division events were observed occasionally in the *minD* mutant. The wild-type strain (DK5133) and a *minD* mutant (DK5155) were used to generate all of the data presented in this figure.

Mother cells of the *minD* mutant, here defined as cells that contain chromosomes and are capable of division, were on average twice as long (8.8 ± 3.4 μm) as the wild type (4.5 ± 1.7 μm) (Fig. 5B), but why the *min* mutant cells were elongated was unclear. One early model suggested that *min* mutants produced elongated cells because divisions that gave rise to minicells came at the expense of medial division events (58, 61). Microscopic analysis, however, indicated that the overall rate of division (counting both medial and polar events) in the *minD* mutant was slightly higher than that seen with the wild type (Fig. 2A). Considering the divisions that gave rise to the different cell types separately, one-quarter of the division events in *minD* gave rise to minicells and three-quarters of the division events occurred along the midcell to produce two mothers. Mother cell division in the *minD* mutant occurred at roughly the same average rate as in the wild type, albeit with a higher standard deviation (Fig. 2B). The wide variance in *minD* midcell division was due to occasional longer-than-average division times that gave rise to very long cells which then experienced shorter-than-average division times with multiple division events per compartment that could occur simultaneously or slightly offset from one another (Fig. 6) (19, 62). We conclude that *min* mother cells experience medial division at approximately the same rate as the wild type, and thus, rate of cellular division does not appear to account for the length differential between *minD* cells and the wild type.

Another factor that could contribute to cell length is the rate of cell elongation, as an elongation rate that is higher than the division rate could give rise to longer cells. Measuring the rate at which the cell poles moved away from one another, the *minD* mutant appeared to elongate more rapidly than the wild type (Fig. 2C). Elongation, however, occurs by the lateral synthesis of cell wall material distributed along the length of the rod (63–65), and thus, the *minD* mutant might appear to elongate more rapidly simply because longer cells have more positions between the poles in which to insert new cell wall material per unit time. Accordingly, the data representing the instantaneous elongation rate, defined as the rate of elongation divided by the length of the cell, were similar for the wild type and the *minD* mutant (Fig. 2D). Moreover, cells of the wild type and the *minD* mutant accumulated biomass at the same rate, as the two strains had indistinguishable growth curves as shown by optical density measurement in broth culture (Fig. 2E). We conclude that the cells of the *minD* mutant elongate at the same rate as those of the wild type; thus, the presence of elongated cells cannot be explained by the rate of elongation.

Another possible explanation for the increased cell size of *min* mutants is a reduced rate at which FtsZ monomers are added to form FtsZ rings. “Adder” hypotheses for cell growth predict that cell size is dictated by the accumulation of a critical threshold amount of a particular cell component, in this case, FtsZ (58, 66–68). To test this hypothesis, we first measured the total amount of mNeongreen-FtsZ per cell and found that *minD* cells actually exhibited higher total fluorescence intensity than the wild-type cells (Fig. 5D). The higher total fluorescence intensity per cell may have been due to the fact that the *minD* cells are longer and maintain multiple Z-rings per compartment. Indeed, when fluorescence intensity was divided by cell length, the *minD* cells and wild-type cells exhibited the same fluorescence density (Fig. 5E). The magnitude of peak FtsZ fluorescence intensity was higher in the wild type than in the *minD* mutant, however (Fig. 5F), suggesting that the local amount of FtsZ may need to accumulate to a certain threshold in order to promote cell division. We infer that the increased cell size of the *minD* mutant is not due to a reduced pool of FtsZ or to a change in the rate of FtsZ synthesis but is instead due to the local amount of FtsZ constituting the Z-rings.

As total FtsZ accumulated at a rate proportional to cell length (Fig. 5E) and as the *minD* mutant constitutively maintained multiple Z rings, new FtsZ monomers synthesized during growth were likely divided between multiple foci, effectively reducing the rate of local FtsZ accumulation that gives rise to cytokinesis. Indeed, the nascent Z-rings of the *minD* mutant exhibited a 2- to 3-fold increase in maturation time (Fig. 4; see also Fig. S2E), likely because the old rings were not dissolved and because each Z-ring independently competed for newly synthesized subunits. We note that the cytokinetic period between FtsZ appearance and septation was 2- to 3-fold longer as well (Fig. 4; see also Fig. S2F), suggesting that regeneration of cell division machinery was also delayed. Paradoxically, the cytokinetic period was substantially longer than the division time (Fig. 4). Each daughter cell of the *minD* mutant, however, was born with at least three persistent Z-rings, including one at each pole and one at the future midcell, and additional Z-rings formed at the one-quarter and three-quarter positions as the cells grew (Fig. 5C). Thus, division at the most central Z-ring allowed daughter cells to be born with a preformed medial ring that would eventually drive septation, and as a result, the medial Z-ring delay time for *minD* had a negative value (Fig. 4; see also Fig. S2D). Despite aberrations in Z-ring parameters, the *minD* mutant still maintained a division time similar to that maintained by the wild type and correlated not with the cytokinetic period but rather with the rate of FtsZ ring appearance (Fig. 4; see also Fig. S2A). We conclude that the Min system disassembles polar Z-rings to recycle and redistribute monomer units, thereby promoting rapid, singular FtsZ accumulation and maturation at a medial site for the proper maintenance of both the cytokinetic period and cell length (Fig. 4).

### The Min system inhibits peptidoglycan turnover, especially at the cell poles.

**FIG 7:**
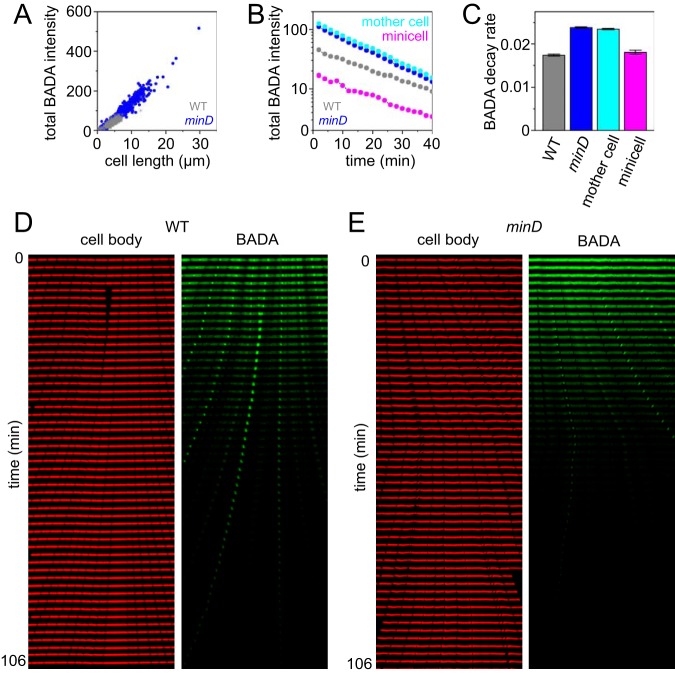
*minD* mutants lose BADA staining fluorescence intensity faster than the wild type. Cells were grown in the presence of the peptidoglycan synthesis/remodeling indicator stain BADA for 4 min, the stain was washed out of the microfluidic device for 8 min, and then fluorescence intensity was tracked in time. (A) A graph of the total BADA intensity per cell per the length of the cell measured in the first frame of the experiment. Each dot represents an individual cell of the wild-type strain (gray) or the *minD* mutant (blue). (B) A graph of the total BADA intensity per cell per unit time after washout of fluorescent d-amino acid. Gray, wild type; blue, *minD* mutant; cyan, mother cells of the *minD* mutant; magenta, minicells of the *minD* mutant. (C) A graph of the rate of decrease in BADA intensity loss as measured by the slope of the lines shown in panel B. (D) A representative kymograph of the wild type after BADA washout. (E) A representative kymograph of the *minD* mutant after BADA washout. The wild-type (DK4393) and *minD* mutant (DK4407) strains were used for the experiments whose results are shown in all panels in this figure. Over 500 measurements were taken for each strain.

Mutants defective in the Min system not only produce longer cells but also produce minicells by division at the cell poles. Peptidoglycan at the cell poles is traditionally considered to be “inert” such that, once synthesized, it experiences little *de novo* synthesis and turnover (65, 69–71). To study how minicells formed, cells in the microfluidic device were presented with a subgenerational (4-min) impulse of a fluorescent d-amino acid that is able to be incorporated into peptidoglycan during either synthesis or remodeling (72, 73). In the wild type, fluorescent peptidoglycan signal was incorporated along the central part of the cell body with peak fluorescence at the nascent division planes (Fig. 1C). Moreover, the troughs of peptidoglycan fluorescence intensity coincided with troughs of cytoplasmic fluorescence intensity consistent with the idea that polar peptidoglycan was less dynamic than at other positions (Fig. 1C). Peptidoglycan staining of the *minD* mutant, however, appeared more intense and more uniform than the staining of the wild type (Fig. 1D). In both the wild type and the *minD* mutant, peptidoglycan staining was proportional to cell length, but the *minD* mutant accumulated more stain per micrometer (Fig. 7A). Also, unlike the wild type, troughs in cytoplasmic staining intensity in the *minD* mutant did not necessarily correspond to decreases in peptidoglycan staining at the cell poles (Fig. 1D). We conclude that the Min system inhibits peptidoglycan synthesis/remodeling and is an important factor in making the polar peptidoglycan appear inert.

We next wanted to determine how the *minD* mutant appeared to accumulate more fluorescent peptidoglycan signal. As described above, cells were stained with a 4-min pulse of fluorescent d-amino acid and washed out with fresh LB medium such that the fluorescence signal would be gradually lost during growth (Movie S5 and S6). When the amount of fluorescence signal per cell per unit time was projected, mother cells of the *minD* mutant experienced a higher overall rate of fluorescence decay than the wild-type or *minD* mutant minicells (Fig. 7B and C; see also Fig. S3A and B). Consistent with inert polar peptidoglycan in the wild type, the most intense staining occurred at the nascent division plane, which, upon becoming a cell pole, remained fluorescent for an extended period of time (Fig. 7D). The *minD* mutant, however, did not exhibit intense staining at the division plane, and polar staining persisted primarily in minicells (Fig. 7E). We conclude that the Min system, at least in B. subtilis, appears to restrict peptidoglycan remodeling throughout the cell but has the greatest effect at the cell poles.

10.1128/mBio.03197-19.3 FIG S3 The *minD* mutant accumulates and loses fluorescent BADA signal more rapidly than the wild type. (A) Total BADA fluorescence intensity after a 4-min staining impulse and washout of the BADA stain in the wild type (DK4393). Points represent averages, and whiskers represent standard deviations of over 500 measurements. (B) Total BADA fluorescence intensity after a 4-min staining impulse and washout of the BADA stain in the *minD* mutant (DK4407). Points represent averages, and whiskers represent standard deviations of over 500 measurements. Cyan indicates measurements of the mother cells, and magenta indicates measurements of minicells. Download FIG S3, PDF file, 0.7 MB. Copyright © 2020 Yu et al. 2020 Yu et al. This content is distributed under the terms of the Creative Commons Attribution 4.0 International license.

10.1128/mBio.03197-19.9 MOVIE S5 Wild-type growth in microfluidic channels with fluorescent BADA, which stains newly synthesized/remodeled peptidoglycan. Constitutive cytoplasmic mCherry is falsely colored red, and staining was performed with a 4-min pulse of BADA (falsely colored green). Images represent strain DK4393. BADA was washed out of the channel for 8 min, after which imaging was recommenced. Movies are 60 frames taken over 120 min at a rate of 1 frame/2 min. Download Movie S5, AVI file, 0.9 MB. Copyright © 2020 Yu et al. 2020 Yu et al. This content is distributed under the terms of the Creative Commons Attribution 4.0 International license.

**FIG 8:**
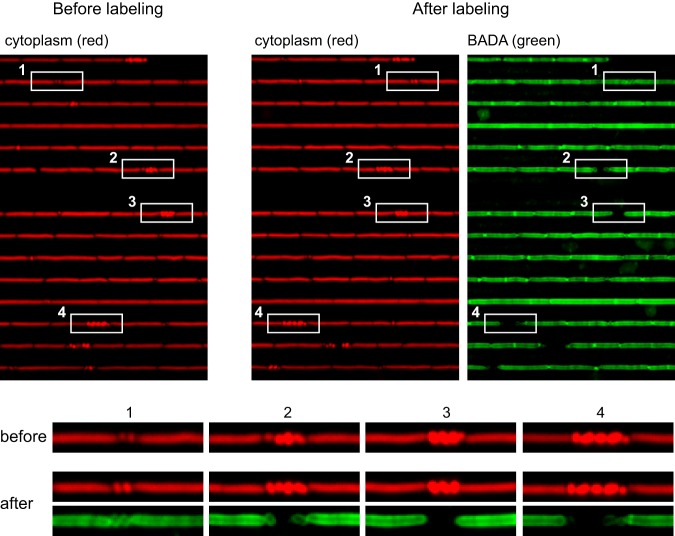
Recently formed minicells are proficient in remodeling of the polar peptidoglycan. (Top panels) Microfluidic analysis of a *minD* mutant (DK4407) expressing cytoplasmic mCherry (falsely colored red; left) and stained for 20 min with the fluorescent d-amino acid BADA (falsely colored green; right). Individual areas of the channels are highlighted by boxes and numbered. (Bottom) Images of the boxes (enlarged to increase detail) with corresponding numbers.

Minicells are thought to be physiologically similar to wild-type cells due to inheritance of cytoplasmic and membrane proteins, but they lack chromosomes and do not grow. One conjecture regarding the fact that the minicells do not grow was that they were thought to be surrounded exclusively by inert polar peptidoglycan, but poles of the *minD* mutant cells appeared much less inert than those of the wild-type cells (Fig. 1D). In cases where minicells formed after the washing out of fluorescent d-amino acid, the new pole of the minicell was unlabeled, but peptidoglycan turnover in the minicell rapidly redistributed circumferential staining (Fig. S4). To further explore the dynamics of polar peptidoglycan observed in the *minD* mutant, the mutant was stained for a longer period of time (20 min). Prolonged exposure of the *minD* mutant to fluorescent d-amino acid resulted in staining of minicell peptidoglycan but only in the most recently formed minicells (Fig. 8). Little to no fluorescence was observed in older minicells, suggesting that the minicells rapidly lost their ability to remodel peptidoglycan (Fig. 8). We conclude that minicells transiently retain the ability to remodel peptidoglycan and that the rapid loss of remodeling capacity may be responsible for the inability of minicells to grow. Combined, our data from microfluidic analyses indicate that the Min system in B. subtilis is multifunctional: Min not only disassembles polar FtsZ-rings but also restricts polar peptidoglycan remodeling.

10.1128/mBio.03197-19.4 FIG S4 A minicell redistributed peptidoglycan stain from its old cell pole to its new cell pole. Cells were stained with BADA for 4 min, and then medium lacking BADA was introduced to wash out the dye, and a kymograph of one channel was generated. Here, we highlight an instance in which a minicell was formed by polar division soon after the dye had been removed such that old pole was stained but the division plane and resulting new pole were not (white caret). Over time, the fluorescent signal incorporated into the peptidoglycan becomes uniformly redistributed around the circumference of the minicell (black caret). Download FIG S4, PDF file, 0.7 MB. Copyright © 2020 Yu et al. 2020 Yu et al. This content is distributed under the terms of the Creative Commons Attribution 4.0 International license.

## DISCUSSION

Binary fission of rod-shaped bacteria is considered one of the simplest forms of cell division, but it is nonetheless complex. Cells elongate by directing envelope synthesis parallel to the long axis of the cell but must periodically reorient envelope synthesis perpendicularly to the long axis to promote cytokinesis. Perpendicular peptidoglycan synthesis is directed by FtsZ, which forms a ring-like scaffold to recruit the cytokinetic complex, and the Min system is thought to help guide FtsZ localization. Here, we quantitatively measured growth and FtsZ dynamics in wild-type cells and *minD* mutant cells of B. subtilis with a microfluidic device and fluorescence microscopy. We found that the primary functions of the Min system are to disassemble FtsZ rings and recycle FtsZ monomers. A failure to recycle FtsZ monomers has a number of consequences for FtsZ dynamics, including the constitutive maintenance of multiple Z-rings per compartment and a longer cytokinetic period. Despite the alternations in FtsZ dynamics, however, we found that the *minD* mother cells have cell division times similar to that of wild-type cells. Moreover, the *min* mutants are so named because they produce small anucleate minicells made exclusively of polar peptidoglycan thought to be relatively inert. Here, we provide evidence that the Min system is responsible for inhibiting polar peptidoglycan turnover and that minicells transiently retain peptidoglycan remodeling capacity.

The function of the Min system is often described as promotion of medial Z-ring formation by preventing FtsZ-ring formation at the poles, but our data, along with data reported previously by others, suggest that, at least in B. subtilis, the primary function is in FtsZ-ring disassembly (23, 30, 35, 46, 60, 74). For example, the Min system does not seem necessary to occlude polar FtsZ-ring formation in B. subtilis, as spore outgrowth experiments showed that in the first generation, FtsZ preferentially localizes to the midcell even in a *minD* mutant (75, 76). Moreover, overproduction of FtsZ in E. coli produces minicells in the presence of an intact Min system, suggesting that the Min system does not formally occlude polar Z-ring assembly (77). The idea of a disassembly function is also supported by genetic evidence showing that mutants with enhanced Z-ring stability are resistant to the effects of MinCD and that mutants with reduced Z-ring stability have increased sensitivity (33, 78, 79). That FtsZ disassembly may require a specific factor may also be consistent with the division of Streptococcus pneumoniae, which naturally lacks the Min system and produces Z-foci that are not depolymerized. Z-foci of S. pneumoniae, instead, continually treadmill from the old division site to the new (71). How MinC antagonizes Z-rings when bound to FtsZ is unknown. At the biochemical level, FtsZ binds a molecule of GTP between the subunits in each adjacent pair, and whereas GTP hydrolysis is correlated with protofilament disassembly, MinC does not appear to alter the GTPase activity (7, 23, 80, 81). Some models suggest that either MinC may block protofilament polymerization while disassembly proceeds through treadmilling or MinC may bind and prevent the protofilament bundling necessary for Z-ring stability (9, 10, 31, 82, 83). In any case, our observation that the Min system primarily promotes Z-ring disassembly does not preclude the prevention of FtsZ-ring synthesis as the two are inherently coupled and difficult to distinguish.

The inability to disassemble Z-rings and recycle FtsZ monomers also explains cell length defects associated with *min* mutants. *min* mutants have long been observed not only to produce minicells but also to have mother cells that are longer than those of the wild type (19, 21, 22, 58). Here, we show that the elongation of the *min* mutants was not due to an extended division time or increased elongation rate but rather to the dynamics of local FtsZ accumulation. In the absence of Min, the persistence of the Z-ring becomes effectively infinite, and the cells maintain multiple Z-rings per compartment. Because the density of FtsZ is constant, each Z-ring is forced to compete for newly synthesized FtsZ monomers. Thus, the 2- to 3-fold increase in cell length was correlated with a 2- to 3-fold increase in Z-ring maturation time. Our results are consistent with the “adder” hypothesis for growth and cell length control, as *min* mutant cells must become longer to achieve a critical threshold amount of FtsZ within a Z-ring to promote cell division (58, 66–68). Additionally, we observed that the *minD* mutant showed a 2- to 3-fold increase in the cytokinetic period. We conclude not only that there was a failure to recycle FtsZ but also that cell division machinery was cosequestered at latent Z-rings and also required *de novo* synthesis for regeneration (74).

Min-mediated disassembly of the latent polar Z-ring coincided with rapid accumulation of a medial Z-ring in the wild-type cell division cycle. Despite defects in FtsZ dynamics, the *minD* mutant did not suffer a defect in either growth rate or division time, suggesting that the cell cycle was robust and depended on another factor. In the absence of Min, the cytokinetic period exceeded the cell division time, but the Z-ring formation that would give rise to the future division was initiated in the preceding generation. Thus, similarly to the manner in which multifork replication allows daughters to inherit partially replicated chromosomes and grow at a rate higher than that seen in the replication period, *minD* daughter cells inherit a mature Z-ring to complete cell division on time and assemble two new Z-rings for the next generation. Ultimately, the division time of the *min* mutant is governed by the rate of Z-ring appearance, but what governs the Z-ring appearance rate is unclear. One likely regulator of Z-rings is the chromosome because Z-rings are prevented from forming over the mass of the genetic material by nucleoid occlusion (84, 85). We infer that the cell division cycle is preserved because the regular period of chromosome replication and segregation dictates the rate at which Z-rings appear, and we note that the Z-ring appearance period is similar to the replication period reported in B. subtilis (56, 86, 87).

The failure to disassemble Z-rings in the absence of Min leaves behind a preformed polar Z-ring that can give rise to polar cytokinesis and results in the classic phenotype of minicell division. We note, however, that despite the fact that each daughter inherits three Z-rings, two at the pole and one at the midcell, and that each Z-ring sequesters division machinery, the division events that gave rise to minicells were nonetheless less frequent. Competition between the Z-rings for newly synthesized FtsZ monomers and divisome components appears to represent inequality, such that the medial Z-ring is stochastically favored. We suspect that the medial rings are favored because they are flanked by chromosomes on either side, each expressing divisome components, and that polar Z-rings are disfavored by diffusion, being proximal to only one chromosome. Consistent with a positional bias in diffusion and capture, medial divisions occur at approximately twice the frequency of minicell divisions, and we found that minicell formation at either the new or old pole was equally probable (Fig. 2F). Regardless of the side of the cell on which polar division occurs, the minicell compartment is reduced to a sphere in which half of the peptidoglycan comes from the preexisting pole and half comes from the nascent septation event.

Polar peptidoglycan of rod-shaped cells has traditionally been considered to be inert such that reduced rates of remodeling relative to the length of the cell are experienced. What makes poles behave as though they are inert, however, is unknown. Here, we used a dye that stains peptidoglycan during either synthesis or remodeling to show that in the absence of Min, polar peptidoglycan is indistinguishable from the rest of the cell. How MinD, a protein in the cytoplasm, would inhibit peptidoglycan extracellular remodeling is unclear, but we note the B. subtilis Min system interacts with a polarly localized multipass transmembrane protein, MinJ (43, 44). MinJ has recently been shown to interact with RodZ, a protein involved in peptidoglycan synthesis/remodeling, and perhaps the MinCDJ complex keeps RodZ away from the poles (88–91). Moreover, why minicells fail to grow is poorly understood, but perhaps it is due to the fact that they quickly lose the ability to remodel their peptidoglycan. The loss of remodeling capacity could be due to the instability of a single protein and its corresponding transcript, but we note that the most substantial difference between mother cells and minicells of a *minD* mutant is the absence of the chromosome. Thus, the chromosome not only directs the cell division cycle but also directly or indirectly dictates cell envelope remodeling, and we note that RodZ is a transmembrane protein with a predicted cytoplasmic DNA binding domain (56, 88, 90, 92).

## MATERIALS AND METHODS

### Strains and growth conditions.

Bacillus subtilis strains (Table 1) were grown in lysogeny broth (LB) (10 g tryptone, 5 g yeast extract, 10 g NaCl per liter) or on LB plates fortified with 1.5% Bacto agar at 37˚C. Antibiotics were supplemented when appropriate at following concentrations: 100 μg/ml spectinomycin, 5 μg/ml kanamycin, 10 μg/ml tetracycline, 5 μg/ml chloramphenicol, and macrolide-lincosamide-streptogramin B (MLS; 1 μg/ml erythromycin, 25 μg/ml lincomycin). For *P_hyspank_* promoter-dependent gene expression, 1 mM isopropyl-β-d-1-thiogalactopyranoside (IPTG) was added to the medium.

**TABLE 1:** Strains

Strain	Genotype
DK4393	*Δhag ΔepsE amyE*::*P_hyspank_-mCherry spec*
DK4407	*Δhag ΔepsE amyE*::*P_hyspank_-mCherry spec minD*::Tn*YLB kan*
DK5133	*ΔepsH mNeongreen-ftsZ amyE*::*P_hyspank_-mCherry spec*
DK5155	*ΔepsH mNeongreen-ftsZ amyE*::*P_hyspank_-mCherry spec minD*::Tn*YLB kan*
DS3187	*ΔminJ minD*::Tn*YLB kan amyE*::*P_hag_-hag^T209C^ spec*

### Strain construction.

All strains were generated in a derivative of B. subtilis ancestral strain 3610 with enhanced frequency of natural competence for DNA uptake, namely, strain DK1042 (93). Cells were mutated for the production of extracellular polysaccharide (EPS) to prevent biofilm formation within the microfluidic device by in-frame markerless deletion of either the *epsE* gene or the *epsH* gene, encoding enzymes essential for EPS biosynthesis, as described previously (94, 95). Some strains were mutated for flagellar biosynthesis to prevent motility within the microfluidic device by an in-frame markerless deletion of the *hag* gene, encoding the flagellar filament protein Hag, as described previously (94). Note that we eventually found that disruption of motility was unnecessary for microfluidic analysis. The mNeongreen fluorescent fusion to FtsZ, a generous gift of Ethan Garner (Harvard University), was crossed into the indicated genetic background by SPP1 phage-mediated transduction (96), and the antibiotic resistance cassette was eliminated as described previously (9). The *minD* gene was mutated in some strains by SPP1 phage-mediated transduction of a transposon mutant allele from DS3187 (*minD*::Tn*YLB-1 kan* transposon insertion in the middle of the *minD* gene at sequence tag TAAATAGAG) as reported previously (44). Finally, the *P_hyspank_-mcherry* inducible construct was introduced by integration of pEV6 at the *amyE* locus and selection for spectinomycin resistance (97).

### Microfluidic system.

A microfluidic device was fabricated through a combination of electron beam (e-beam) lithography, contact photolithography, and polymer casting (52). Briefly, the microfluidic device is comprised of fluid and control layers both cast in poly(dimethylsiloxane) (PDMS) and a glass coverslip. The fluid layer lies between the control layer and glass coverslip and contains the microchannels and channel array to trap the bacteria. Media and cells are pumped through the microfluidic channels by on-chip peristaltic pumps and valves that are controlled pneumatically through the top control layer. Each pneumatic valve is controlled by software to apply either vacuum (0.3 bar) or pressure (1.3 bars) to open or close individual valves, respectively. Device design details are included in Fig. S1 in the supplemental material.

### Microfluidic device materials.

We purchased glass slides (50 mm by 75 mm) from Corning, Inc.; poly(dimethylsiloxane) (PDMS, Sylgard 184) from Dow Corning, Inc.; No. 1.5 Gold Seal coverslips (48 mm by 60 mm) from VWR International, LLC; SU-8 2010 photoresist and SU-8 developer from MicroChem Corp.; titanium diisopropoxide bis(2,4-pentanedionate) from Gelest, Inc.; syringe filters (0.22-μm pore size) from Wheaton Company; and all other chemicals from Sigma-Aldrich Co.

### Microfluidic device fabrication.

Control and fluid layer masters were fabricated by a combination of electron-beam (e-beam) lithography and contact photolithography in negative-tone photoresist SU-8. Glass slides were cleaned in a soap solution (Branson) with sonication, rinsed with water, soaked in a SC-1 clean solution (hydrogen peroxide, ammonium hydroxide, deionized (DI) water, 75°C) to remove any organic contaminants, rinsed with water, dried with nitrogen, and dehydrated on a hot plate at 150°C for 30 min. A resist adhesion promoter of 2% titanium diispropoxide (bis-2,4-pentanedionate) in isopropanol was spin coated on the clean glass slides, and a 20-μm-thick base layer of SU-8 2010 was immediately spin coated onto the glass substrates. The glass substrates were prebaked at 95°C for 4 min and exposed to 225 mJ/cm^2^ of UV light with an exposure system (205S; Optical Associates, Inc.). After exposure, the substrate was postbaked at 95°C for 5 min.

Nanochannel arrays were fabricated by diluting the SU-8 photoresist 8% to 34% (wt/vol) in cyclopentanone, spin coating a 1.0-μm-thick layer onto the base layer, and prebaking at 95°C for 1 min. A scanning electron microscope (SEM) (Quanta 600F; FEI Company) equipped with a nanometer pattern generation system (JC Nabity Lithography Systems) was used to create the nanochannels in the SU-8 resist. An electron beam area dose of 1.5 μC/cm^2^ was used at a 30-kV accelerating potential to expose the nanochannel pattern, which consisted of 600 channels written in a 5-by-4 grid, where each section of the grid was composed of 30 channels. After e-beam exposure, the substrates were postbaked at 95°C for 2 min and developed in SU-8 developer for 1 min. To create the microchannels in an orientation orthogonal to the nanochannel arrays, an additional 20-μm layer of SU-8 2010 resist was spin coated over the nanochannels. After a prebake was performed at 95°C for 6 min, the SU-8 substrates were exposed to 225 mJ/cm^2^ of UV light through the fluid-layer photomask (International Phototool Company, LLC). The substrates were then postbaked for 7 min and developed for 2 min in SU-8 developer.

Microchannels on the control layer master were fabricated in a 40-μm-thick layer of SU-8 2010 photoresist exposed to 350 mJ/cm^2^ of UV light through the control-layer photomask (International Phototool Company, LLC). After exposure, the substrates were postbaked for 9 min and developed for 5 min in SU-8 developer. To ensure a clean and repeatable cast of PDMS from the SU-8 masters, the masters were coated with (tridecafluoro-1,1,2,2-tetrahydrooctyl) trichlorosilane by vapor deposition in a desiccator overnight.

PDMS replicas of the control layer were fabricated by creating a tape boundary around the control-layer master and dispensing 8 ml of uncured PDMS (10:1) onto the masters. The PDMS was degassed under vacuum for 30 min and cured at 70°C in the oven for 1 h. After the tape boundary was removed, PDMS replicas were peeled off the masters and trimmed to fit the fluid layer. Holes were punched through pneumatic access ports with a 2.5-mm-diameter biopsy punch. To create the PDMS replicas of the fluid layer, 3 ml of uncured PDMS (10:1) was spread onto the master and degassed under vacuum for 1 h. The substrates were then spun at 1,000 rpm for 30 s to form an ∼100-μm-thick layer of PDMS. After being allowed to rest for 1 h, the fluid layer was partially cured at 70°C for 10 min. The control layer was aligned with the fluid layer under a stereoscope (Leica, Inc.), and the two layers were brought together and cured at 70°C overnight to ensure a strong bond between them. The bonded control and fluid layers were carefully peeled off the master, and fluid input/outlet holes were punched through two layers with 1.0-mm-diameter biopsy punch.

Prior to the step when bonding to glass slides was performed, all valves and pumps in the fluid-layer PDMS were covered with plastic masks to prevent irreversible adhesion. The bonded control and fluid layers and glass coverslip were plasma treated (PDC-32G; Harrick) for 90 s. The plastic masks were removed, and the PDMS surface of the fluid layer was immediately brought into contact with the glass coverslip to form an irreversible bond.

### On-device cell culture.

Prior to loading cells into the microfluidic device, the fluidic channels were coated with 1% bovine serum albumin (BSA) for 1 h to act as a passivation layer. Then, all the channels were filled with 1 mM IPTG–0.1% BSA–LB medium (10 g tryptone, 5 g yeast extract, 10 g NaCl per liter). A saturated culture of cells (∼25 μl) was added through the cell reservoir and pumped into the cell-trapping region. During cell loading, vacuum was applied to the control layer above to open the membrane region. After a sufficient number of cells were pumped underneath the channel array, positive pressure was applied to trap individual cells in those channels. Medium was pumped through the microchannels to flush away excess cells. After the excess cells were pumped away, medium was continuously flowed through the microchannels by gravity flow during the entire experiment.

### Time-lapse image acquisition.

Steady-state cell growth was monitored from 3 to 21 h postinoculation. Fluorescence microscopy was performed on a Nikon Eclipse Ti-E microscope and an Olympus IX83 microscope. The Nikon Eclipse Ti-E microscope was equipped with a 100× Plan Apo lambda, phase-contrast, 1.45-numerical-aperture (NA), oil immersion lens objective and a Photometrics Prime95B scientific complementary metal oxide semiconductor (sCMOS) camera with Nikon Elements software (Nikon, Inc.). Fluorescence signals from mCherry and mNeongreen were captured from a Lumencor SpectraX light engine with matched Chroma mCherry and yellow fluorescent protein (YFP) filter sets, respectively. The Olympus IX83 microscope was equipped with an Olympus UApo N 100×/1.49-NA oil lens objective and a Hamamatsu electron multiplying–charge-coupled-device (EM-CCD) digital camera operated with MetaMorph Advanced software. Fluorescence signal from mCherry was captured from an Olympus U-HGLGPS fluorescence light source with matched tetramethyl rhodamine isocyanate (TRITC) Semrock filter, and signals from mNeongreen and the fluorescent d-amino acid BADA were captured with the same light source and a green fluorescent protein (GFP) filter. Images were captured from at least eight fields of view at 2-min intervals. The channel array was maintained at 37°C with a TC-1-100s temperature controller (Bioscience Tools). For all direct comparisons, the same microscope and settings were used.

### Data analysis.

A period of adaptation following exposure to illumination was observed; thus, data analysis was restricted to periods of steady state. Cell identification and tracking were analyzed by a series of MATLAB programs (The MathWorks, Inc.) (52). The program extracted fluorescence intensity data along a line profile down the longitudinal center of each submicron channel. The cytoplasmic mCherry line profile showed a flat-topped peak on the line where a cell was located, and a local 20% decrease in fluorescence intensity was used to identify cell boundaries after division. Division events were conservatively measured as the time at which one cell became two according to the decrease in fluorescence intensity as described above. Moreover, cell bodies were tracked from frame to frame in order to construct lineages of cell division, and cell body intensity was determined by the integration of the cytoplasmic mCherry signal intensity within the confines of the cell. The mNeongreen-FtsZ signal was similarly tracked and measured along the length of the cell. The FtsZ line profile was normalized by cell body intensity in order to minimize intensity differences across frames and different fields of view.

### Fluorescent d-amino acid labeling.

The fluorescent d-amino acid BADA was supplied by the M. VanNieuwenhze and Y. Brun laboratories. To create BADA, 3-amino-d-alanine was conjugated to BODIPY-FL (72, 73). Stock solutions of BADA (100 mM) were prepared in dimethyl sulfoxide (DMSO) as the reaction mixtures were poorly soluble in water. The stock solutions were then diluted with LB medium to 1 mM BADA with <2% (vol/vol) DMSO left. To label the cells at 3 h postinoculation, the BADA solution (1 mM) was continually pumped through the channel array for 4 min. Excess dye was washed away by pumping LB medium through the channel array for 8 min. Fluorescent images were captured at 2-min intervals from at least eight fields of view.

10.1128/mBio.03197-19.10 MOVIE S6 *minD* growth in microfluidic channels with fluorescent BADA, which stains newly synthesized/remodeled peptidoglycan. Constitutive cytoplasmic mCherry is falsely colored red, and staining was performed with a 4-min pulse of BADA, falsely colored green. Images represent strain DK4407. BADA was washed out of the channel for 8 min, after which imaging was recommenced. Movies are 60 frames taken over 120 min at a rate of 1 frame/2 min. Download Movie S6, AVI file, 0.9 MB. Copyright © 2020 Yu et al. 2020 Yu et al. This content is distributed under the terms of the Creative Commons Attribution 4.0 International license.
