# Supplementary figures and images for: The Min System Disassembles FtsZ Foci and Inhibits Polar Peptidoglycan Remodeling in Bacillus subtilis
Source: mBio. 2020 Mar 17;11(2):e03197-19. doi: 10.1128/mBio.03197-19 (PMC7078482; doi:10.1128/mBio.03197-19)

Figure S1

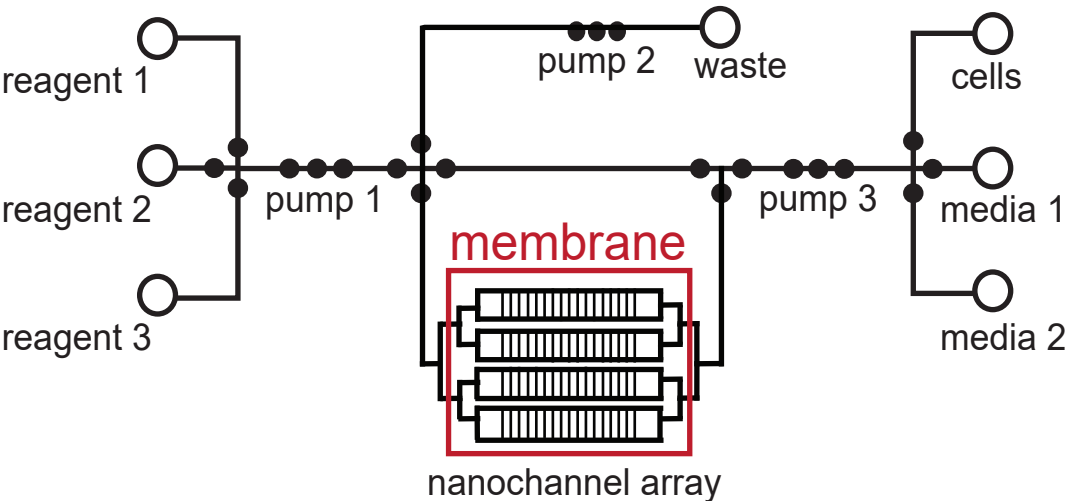

Supplement: FIG S1 [file mBio.03197-19-sf001.pdf]

Figure S2

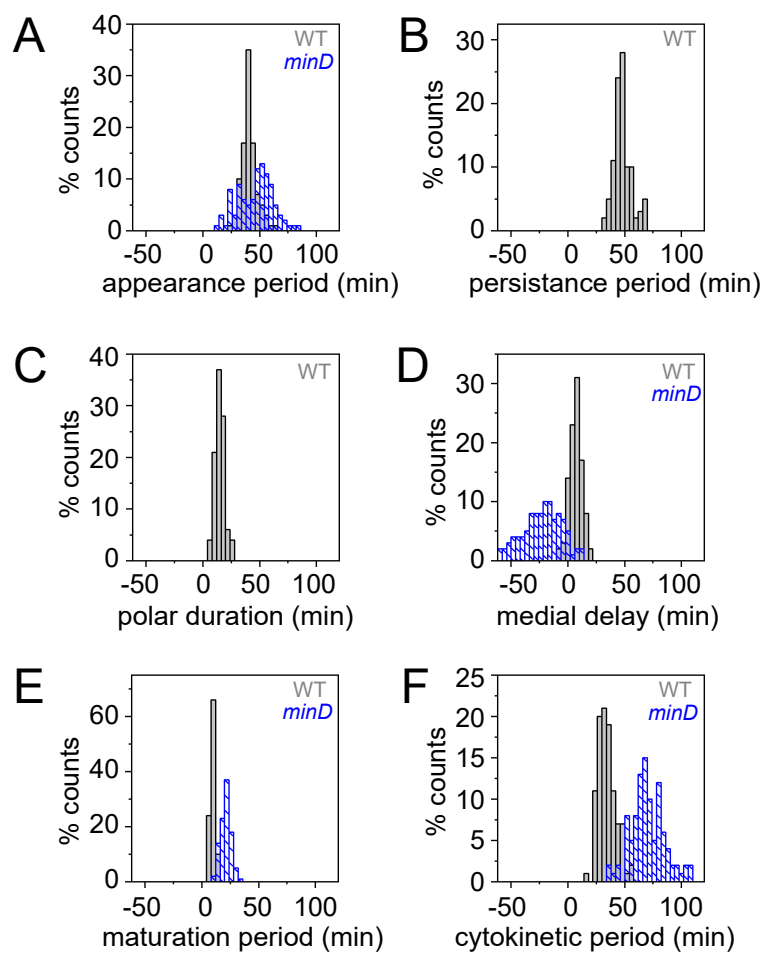

Supplement: FIG S2 [file mBio.03197-19-sf002.pdf]

Figure S3

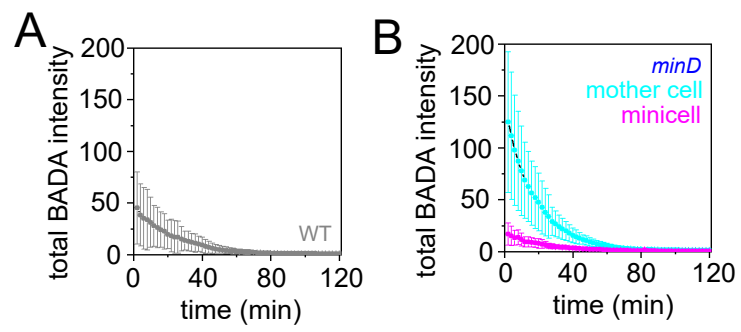

Supplement: FIG S3 [file mBio.03197-19-sf003.pdf]

Figure S4

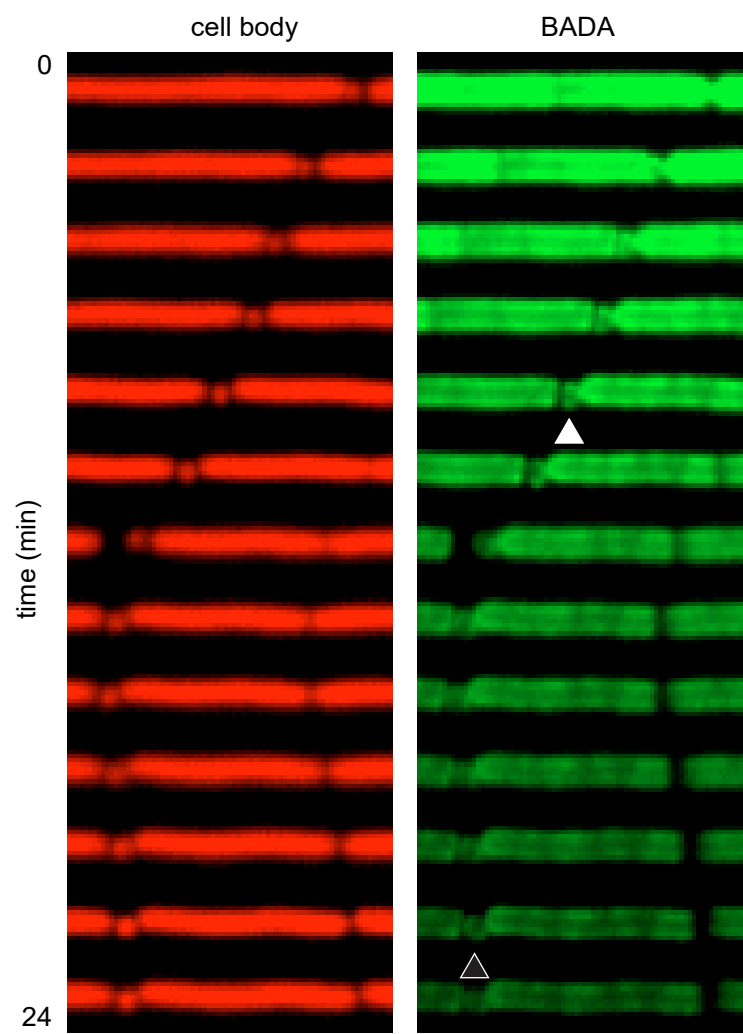

Supplement: FIG S4 [file mBio.03197-19-sf004.pdf]
